# Acute and Chronic Toxicity of an Aqueous Fraction of the Stem Bark of *Stryphnodendron adstringens* (Barbatimão) in Rodents

**DOI:** 10.1155/2013/841580

**Published:** 2013-07-18

**Authors:** Marco Antonio Costa, João Carlos Palazzo de Mello, Edílson Nobuyoshi Kaneshima, Tânia Ueda-Nakamura, Benedito Prado Dias Filho, Elisabeth Aparecida Audi, Celso Vataru Nakamura

**Affiliations:** ^1^Programa de Pós-Graduação em Ciências Farmacêuticas, Universidade Estadual de Maringá, Avenida Colombo 5790, 87020-900 Maringá, PR, Brazil; ^2^Departamento de Farmácia, Universidade Estadual de Maringá, Avenida Colombo 5790, 87020-900 Maringá, PR, Brazil; ^3^Departamento de Ciências Básicas da Saúde, Universidade Estadual de Maringá, Avenida Colombo 5790, 87020-900 Maringá, PR, Brazil; ^4^Departamento de Farmacologia e Terapêutica, Universidade Estadual de Maringá, Avenida Colombo 5790, 87020-900 Maringá, PR, Brazil; ^5^Laboratório de Inovação Tecnológica no Desenvolvimento de Fármacos e Cosméticos, Avenida Colombo 5790, Bloco B08, 87020-900 Maringá, PR, Brazil

## Abstract

*Stryphnodendron adstringens* has a high tannin content and is used as an antiseptic and antimicrobial and in the treatment of leucorrhea, gonorrhea, wound healing, and gastritis. The present study evaluated the toxic effects of the heptamer prodelphinidin (F2) from the stem bark of *S. adstringens* in rodents. In the acute toxicity test, the mice that received oral doses exhibited reversible effects, with an LD_50_ of 3.015 mg · kg^−1^. In the chronic toxicity test at 90 days, Wistar rats were treated with different doses of F2 (10, 100, and 200 mg · kg^−1^). In the biochemical, hematological, and histopathological examinations and open-field test, the different dose groups did not exhibit significant differences compared with controls. The present results indicate that F2 from the stem bark of *S. adstringens* caused no toxicity with acute and chronic oral treatment in rodents at the doses administered.

## 1. Introduction

The bioactivity of various compounds in medicinal plants has been assessed. These compounds are isolated and analyzed to determine biological activity, mechanisms of action, and toxicity [1, 2]. The genus *Stryphnodendron* sp belongs to the family Fabaceae (native savanna), approximately 48 species of which have been identified, including *Stryphnodendron adstringens* (Mart.) Coville (known as “barbatimão”). This species is found in the central savannah region of Brazil [3–5]. This plant is popularly used as an antiseptic and antimicrobial and in the treatment of leucorrhea, gonorrhea, gastritis, diarrhea, bleeding, and wound healing [6–9]. Scientific research has shown that it has antiulcerogenic potential, antiprotozoan activity, anti-inflammatory effects, antimicrobial activity, and wound healing effects [6, 10–18].

The bark of *S. adstringens* is rich in proanthocyanidin polymers, including several flavan-3-ols, such as prodelphinidins and prorobinetinidins [19–22]. The chemical composition of prodelphinidin (F2) has been partially defined as a heptamer compound [16].

The toxicity of *S. adstringens* was the subject of a study by Rebecca et al. [23, 24]. The crude extract of stem bark was administered at high doses in mice and tested in liver mitochondria, showing signs of liver toxicity. Other studies have been conducted with other parts of the plant [25, 26]. Studies by De Sousa et al. [27] and Costa et al. [28] showed that *S. adstringens* had no genotoxic effects in *Drosophila melanogaster* or micronuclei (bone marrow) and *Artemia salina* tests in mice, respectively.

Thus, considering the wide use of this plant and that few studies have been conducted to determine the toxicological profile of *S. adstringens*, the present study sought to obtain more information about toxicity at therapeutic doses.

## 2. Materials and Methods

### 2.1. Plant Material

Stem bark from *Stryphnodendron adstringens* was collected in São Jerônimo da Serra, Paraná, Brazil (S23°43′7.8′′, W50°45′23.5′′; altitude 926 m), in March 2008. A voucher herbarium specimen was deposited at the Universidade Estadual de Maringá (no. HUM 14321).

### 2.2. Aqueous Fraction

The bark was dried at room temperature and then pulverized. The crude extract was obtained by turboextraction of the bark at 1,000 ×g with 70% acetone in water for 15 min. The organic solvent was eliminated by rotavapor and lyophilized to yield a crude extract (F1; 300 g). F1 (50 g) was suspended in water (500 mL) and partitioned with ethyl acetate (500 mL; 1 : 1) to obtain a proanthocyanidin polymer-rich fraction (aqueous fraction; F2; 35 g).

### 2.3. Animals

Adult Wistar rats (90 days old), weighing 230–240 g (female) and 355–365 g (male), and Swiss mice (60 days old), weighing 35–45 g (female) and 45–60 g (male), were used and housed in groups of five per cage, with food and water freely available. The animals were maintained on a 12 h/12 h light/dark cycle under controlled temperature (22 ± 1°C). The protocol was approved by the Ethical Committee of the State University of Maringá (Approval no. 026/2009).

### 2.4. Toxicity Studies

The toxicity studies were performed according to the Brazilian National Health Surveillance Agency (ANVISA) [29–32].

#### 2.4.1. Acute Toxicity Study in Mice

Swiss mice were divided into seven groups, with 10 animals per group (five males and five females). Six groups were orally treated by gavage with different doses of F2 (500, 1,000, 2,000, 3,000, 4,000, and 5,000 mg·kg^−1^). One group that received distilled water was included as a negative control. The volume administered by gavage in the mice was approximately 0.3 mL per animal. Water and food were freely available to the animals. The general behavior and number of survivors were observed at 5 min, 15 min, 30 min, 1 h, 2 h, 4 h, and 24 h and daily thereafter until day 14. Toxicological effects were assessed, including changes in locomotion, respiration, piloerection, diarrhea, drooling, altered muscle tone, hypnosis, convulsions, hyperexcitability, writhing (abdominal constrictions), and mortality (which is expressed as the median lethal dose [LD_50_]) [33]. From the 24th hour until day 14, the weights of the animals were recorded.

At the end of this period, all of the animals were sacrificed. The kidneys, heart, lungs, spleen, and liver were removed, weighed, and evaluated for macroscopic abnormalities. When changes were observed in the autopsies, further histological examination of the organs was performed.

#### 2.4.2. Repeated-Dose Oral Toxicity Study in Rats

Wistar rats were divided into four groups (11 males and 11 females). One group served as the control and received only water. The other groups received F2 of *S. adstringens* (10, 100, or 200 mg·kg^−1^) suspended in water and administered orally by gavage daily for 90 days. The volume administered by gavage in the rats was approximately 0.5 mL per animal. The choice of the doses was based on the estimated oral dose in popular use (10 mg·kg^−1^) [34] and 10- and 20-times the effective dose [29, 30].

All of the animals received food and water *ad libitum* during the treatment. They were observed daily with regard to behavior and weighed weekly. At the end of the 90-day period, the animals were deprived of food for 15 h and then sacrificed. Their blood was collected for biochemical and hematological examination. The organs were carefully dissected and removed for weighing, macroscopic examination, and histopathological analysis.

*Blood Analysis*. Biochemical analyses were performed to determine glucose, aspartate aminotransferase (AST), alanine aminotransferase (ALT), alkaline phosphatase (ALP), total protein, creatinine, uric acid, blood urea nitrogen (BUN), triglycerides, total cholesterol, *γ*-glutamyltransferase (*γ*-GT), bilirubin, sodium, and potassium. These were evaluated using the Dimension RXL Max system (Siemens).

Hematological analyses were performed using the automatic counter Pentra 60 ABX (ABX Diagnostics) to evaluate the following parameters: erythrocyte count (red blood cells (RBCs)), hemoglobin, hematocrit, mean corpuscular volume (MCV), mean corpuscular hemoglobin (MCH), mean corpuscular hemoglobin concentration (MCHC), platelet count, and leucocyte count (white blood cells (WBCs)). Differential WBC counts (nonsegmented neutrophils, segmented neutrophils, basophils, eosinophils, lymphocytes, and monocytes) were made using a glass-slide method. Immediately after collection, blood smears were air-dried and stained with Leishman's stain. One hundred cells were randomly counted in each smear, and the percentage of each type was calculated. Any morphological change in the blood cells was noted.

*Organ Weights and Histopathological Analyses*. The organs (thymus, esophagus, stomach, duodenum, lung, heart, kidneys, liver, spleen, adrenals, and sex organs) of all of the animals were examined macroscopically. The positions, shapes, sizes, and colors of the internal organs were visually observed for signs of macroscopic abnormalities. The organs were weighed and fixed in Bouin's fixative and preserved in 70% ethanol. For the 200 mg·kg^−1^ dose of F2 and controls, tissue slides were prepared and stained with hematoxylin and eosin for microscopic examination.

*Open-Field Test (OFT)*. Locomotor behavior was assessed on day 86 of treatment in the open-field test. Each animal was placed in a round wooden arena (70 cm diameter) with 30 cm high walls. Luminosity at the center of the open field was 60 lux during a 5-min period. Rearing, self-cleaning, urination, the number of fecal pellets, and locomotion were recorded [35]. For the evaluation of locomotion, the total distance traveled was analyzed using a video tracking system (Ethovision). 

### 2.5. Statistical Analyses

The results are expressed as mean ± standard deviation (SD). The data were analyzed using Statistica 8.0 software. The statistical analyses were performed using one-way analysis of variance (ANOVA) followed by the Dunnett *post hoc* test. When the variance was not constant, the nonparametric Kruskal-Wallis test was performed to determine significant differences. The histopathological results were analyzed using Fisher's exact test. Differences were considered significant at *P* ≤ 0.05.

## 3. Results

### 3.1. Acute Toxicity Study in Mice

**Figure 1:**
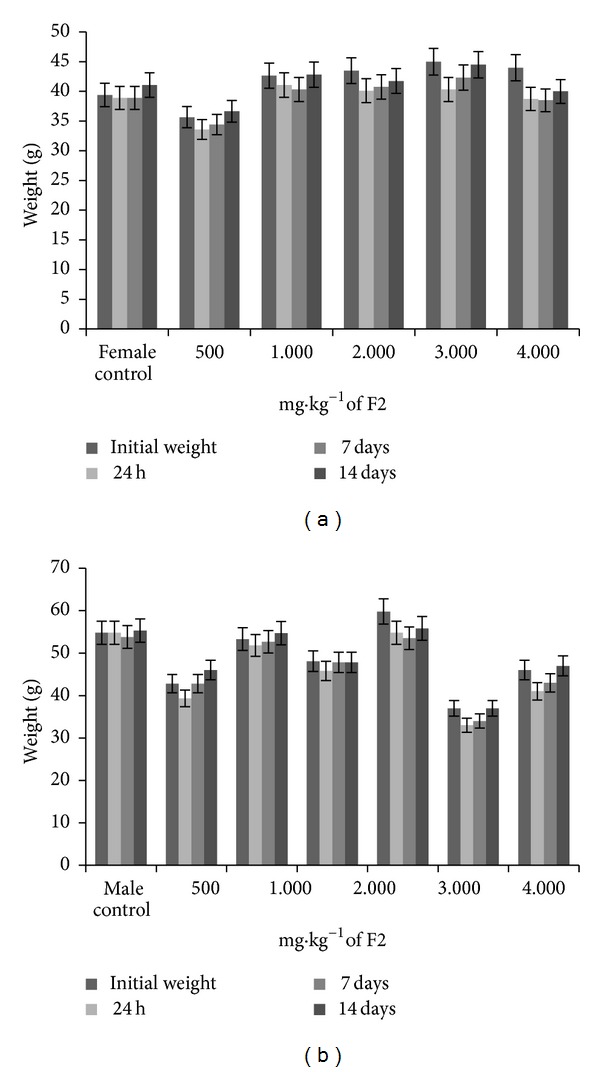
Body weight gain (g) in female mice (a) and male mice (b) treated orally with 500, 1,000, 2,000, 3,000, 4,000, and 5,000 mg·kg^−1^ F2 in the acute toxicity study.

The effects are summarized in Table 1. The doses of 500 and 1,000 mg·kg^−1^ showed no signs of toxicity. Only the 2,000 mg·kg^−1^ dose caused signs of toxicity, beginning with diarrhea and piloerection. At doses of 3,000–5,000 mg·kg^−1^, hypoactivity, hyperventilation, ptosis, hypothermia, motor impairment, sedation, and catatonia were observed, all of which were reversible after 48 h. Death occurred in the groups that received 2,000–5,000 mg·kg^−1^, resulting in an LD_50_ of 3,015 mg·kg^−1^. All of the groups had lower body weights in the first 24 h. After 7 days, however, body weights recovered (Figure 1). With regard to the weights of the organs, only the liver showed a decrease at doses of 4,000 and 5,000 mg·kg^−1^ (Table 2). In the macroscopic and histopathological analyses, no changes were observed at lower doses. Only liver tissue damage was observed at the 5,000 mg·kg^−1^ dose. 

### 3.2. Repeated-Dose Oral Toxicity Study in Rats

#### 3.2.1. Behavior and Body Weight Gain

**Figure 2:**
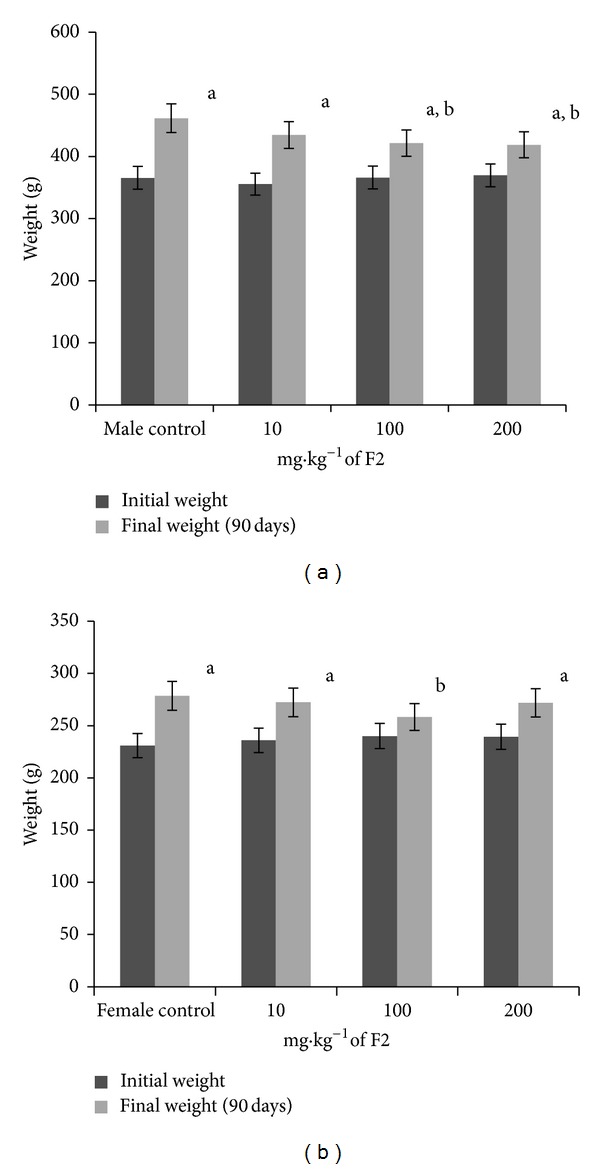
Body weight gain (g) in male rats (a) and female rats (b) treated orally for 90 days with F2 of *S. adstringens* (10, 100, and 200 mg·kg^−1^) and controls. ^a^ *P* < 0.05, compared with initial values; ^b^ *P* < 0.05, compared with control values (ANOVA).

**Figure 3:**
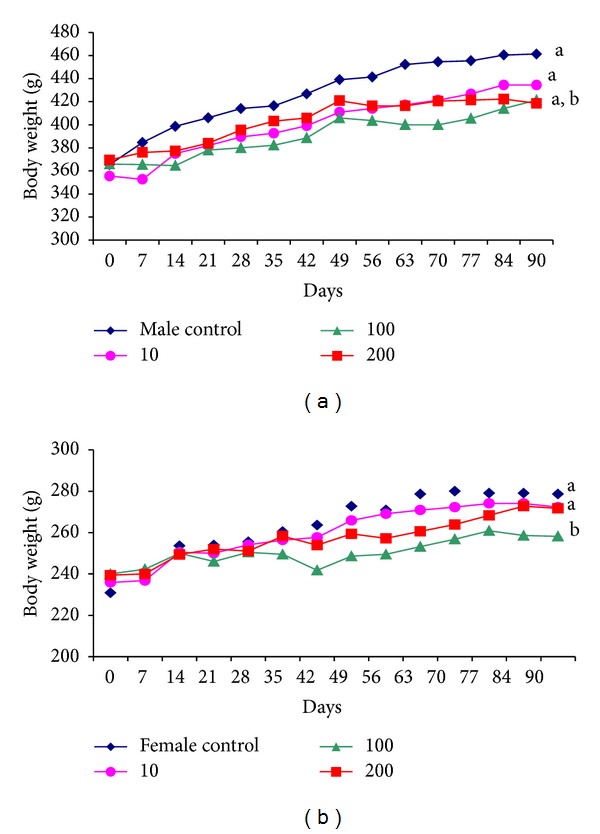
Body weight (g) in male rats (a) and female rats (b) treated orally for 90 days with F2 of *S. adstringens* (10 mg·kg^−1^ ●, 100 mg·kg^−1^ ▲, and 200 mg·kg^−1^ ■) and controls (♦).  ^a^ *P* < 0.05, compared with initial values;  ^b^ *P* < 0.05, compared with controls (ANOVA).

During treatment, no signs of toxicity were observed in the animals. Body weight gain as a function of time is shown in Figures 2 and 3. All of the groups showed weight gain compared with their initial weight, with the exception of the 100 mg·kg^−1^ dose in females. Furthermore, the doses of 100 and 200 mg·kg^−1^ in males and 100 mg·kg^−1^ in females caused less weight gain compared with controls (Figures 2 and 3; Table 3).

#### 3.2.2. Biochemical Analyses

The effects of F2 of *S. adstringens* on biochemical profiles showed specific changes (Table 4). In male rats, some parameters were significantly increased compared with the control group, including potassium (10.7 ± 2.4 mEq·L^−1^ at 100 mg·kg^−1^), uric acid (2.7 ± 1.1 and 2.0 ± 0.8 mg·dL^−1^ at 100 and 200 mg·kg^−1^, resp.), and AST (186.0 ± 47.0 U·L^−1^ at 100 mg·kg^−1^). Other parameters were significantly decreased, including triglyceride (85.5 ± 28.3 mg·dL^−1^ at 200 mg·kg^−1^) and creatinine (0.4 ± 0.1 mg·dL^−1^ at 100 mg·kg^−1^). In female rats, only triglycerides (58.9 ± 12.5 and 72.7 ± 21.3 mg·dL^−1^ at 100 and 200 mg·kg^−1^, resp.) were significantly decreased compared with the control group.

#### 3.2.3. Haematological Analyses

The effects of F2 of *S. adstringens* on hematological parameters showed specific changes but only in male rats (Table 5). Significant differences from the control groups were found, including decreased MCH (20.0 ± 0.4, 19.9 ± 0.5, and 20.0 ± 0.4 pg at 10, 100, and 200 mg·kg^−1^, resp.), decreased MCHC (35.1 ± 0.3% at 100 mg·kg^−1^), and increased platelet counts (929 ± 108 and 961 ± 105 × 10^3^ mm^−3^ at 10 and 100 mg·kg^−1^, resp.). No significant differences were observed in female rats compared with the control group.

#### 3.2.4. Organ Weights and Histopathological Analyses

No macroscopic alterations, differences in relative body weight, or histopathological parameters were observed compared with controls.

#### 3.2.5. Open-Field Test

**Figure 4:**
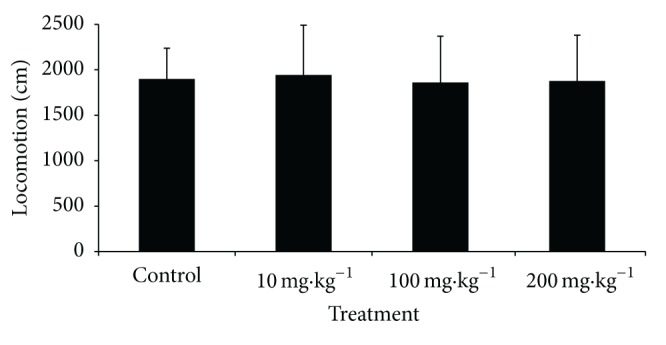
Open-field test results. The figure shows locomotion in rats that orally received F2 of *Stryphnodendron adstringens* at day 86 of treatment. The data are expressed as mean ± SEM. *P < 0.05, compared with control group (ANOVA).

Locomotion (Figure 4), rearing, urination, the number of fecal pellets, and self-cleaning behavior were not significantly different between the treatment groups and controls.

## 4. Discussion

 The indiscriminate use of *S. adstringens* without considering possible toxicity or efficacy can lead to serious health risks. The proanthocyanidin polymer-rich fraction (heptamer compound) of the stem bark of *S. adstringens* (F2) offers possible advantages, including antifungal activity against *Candida* spp., especially *C. albicans* [16]. Vulvovaginal candidiasis is one of the most frequent pathologies observed in daily gynecology practice. Alternative herbal treatments may be less costly and lead to greater adherence to treatment, thus improving the patients quality of life [36–38].

The results of the acute toxicity study indicated that the toxicity of F2 from the stem bark of *S. adstringens* is low (Tables 1 and 2). Some signs of toxicity were observed, which increased progressively with increasing dose but were quickly reversible. No significant changes in organ weight or macroscopic and histological parameters were observed compared with the control group.

The decrease in liver weight at doses of 4,000 and 5,000 mg·kg^−1^ and tissue liver damage at a dose of 5,000 mg·kg^−1^ indicate a direct action of the plant in the liver at high doses, which is consistent with Rebecca et al. [23, 24], who studied doses >800 mg·kg^−1^ in rats. 

 The high LD_50_ values of F2 (3,015 mg·kg^−1^) confirm its low acute toxicity that has been indicated by other parameters [39]. Moreover, Rebecca et al. [23] found an LD_50_ of 2,700 mg·kg^−1^ of the crude extract with acute treatment, demonstrating that the aqueous fraction has less toxicity. The high LD_50_ values indicate that the extract can be administered with a high degree of safety.

In the repeated-dose oral toxicity experiment, no significant changes in behavior or mortality were observed in rats. According to Raza et al. [40], changes in body weight can indicate adverse effects when the animal shows a loss greater than 10% of initial weight. In the present study, however, neither group lost weight during treatment. At doses of 100 and 200 mg·kg^−1^, significant differences in weight gain were observed.

According to Lewis et al. [41], “some factors can be useful in differentiating a significant change from control values, from a treatment-related effect. This difference is less likely to be an effect of treatment if: there is no obvious dose response; it is due to finding(s) in one or more animals that could be considered outlier(s) and it is within normal biological variation (within the range of reference values).”

In the present study, the biochemical results varied widely between doses and between sexes, with no linear profile. The altered values were within normal limits, and the results are considered normal for this animal species. Other values that changed did not show a dose response [42, 43].

In male rats, potassium, AST, and creatinine at 100 mg·kg^−1^, triglycerides at 200 mg·kg^−1^, and uric acid at 100 and 200 mg·kg^−1^ significantly changed. In female rats, only triglycerides at 100 and 200 mg·kg^−1^ significantly changed compared with the control group. To evaluate kidney function, creatinine, urea, sodium, potassium, and uric acid were assessed [44]. Urea and sodium were not different from controls in both female and male rats. In males, significant differences in creatinine and potassium levels in the groups treated with F2 did not show a dose response, and uric acid remained within the normal range (1.2–7.5 mg·dL^−1^) [42]. The same parameters also did not change in females. This suggests that F2 does not adversely affect kidney function, confirmed by the absence of histopathological changes in this organ.

To evaluate liver function, AST, ALT, ALP, total bilirubin, and *γ*-GT were assessed because these are considered markers of liver function, and liver changes have been reported after phytotherapeutic product use [45–48]. In the present study, a significant increase in AST levels was only observed in male rats at the intermediate dose, with no dose response. As noted earlier, the high doses used in the acute study altered liver tissue, which could justify the increase in AST through the release of enzymes from the cells of the damaged organ or changes in cellular membrane permeability [23]. However, this did not appear to be the case at present. Our histopathological examinations did not indicate any cellular lesions.

The levels of total protein, glucose, cholesterol, and triglycerides were measured to assess the general biochemical profile of the animals and determine the presence of metabolic changes. The values were not significantly different between the control and treatment groups in both female and male rats, with the exception of triglycerides, which appeared to be reduced in both females and males but remained within the normal range [43]. High doses of the crude extract of stem bark can cause liver mitochondrion toxicity [23, 24]. Mitochondrial lesions can alter lipid metabolism.

In the present study, hematological changes did not appear to be related to treatment with F2. No changes were observed in females, but changes in MCH (10, 100, and 200 mg·kg^−1^), MCHC (100 mg·kg^−1^), and platelet counts (10 and 100 mg·kg^−1^) were observed in males. However, the changes in MCHC and platelets did not show a dose-response relationship, and the changes in MCH, despite being evident at the three doses tested, remained within the normal range (19.0 ± 1.09) [43]. These results suggest that F2 did not exert effects on blood cells or bone marrow, which are both sensitive to toxicity in animals [49]. This result is supported by the absence of F2 genotoxicity in mice in another study [28].

The relative weights of the organs did not show significant changes in the macroscopic and histopathological examinations in the treatment groups compared with controls in either sex. In contrast to the study by Rebecca et al. [23], which found thymus involution at high doses, lower doses did not affect this organ.

Another interesting result was found in the open-field test, in which none of the parameters showed significant changes, indicating that F2 did not exert neurobehavioral alterations in the animals.

The low toxicity of F2 from the stem bark of* S. adstringens*, reflected by high LD_50_ values, suggests a wide safety margin at therapeutic doses. In the repeated-dose oral toxicity study, no serious signs or significant changes in hematological, biochemical, and histopathological parameters or other remarkable effects were observed in rats. These toxicity studies suggest that the fraction is safe at the doses administered. However, further studies are needed to evaluate other parameters, including carcinogenicity, teratogenicity, and neurotoxicity. Future clinical pharmacology studies should also be conducted to determine tolerance and substantiate its pharmacological use.

## 5. Conclusions

The low toxicity of F2 obtained from stem bark of *S. adstringens* in the acute and repeated-dose oral (chronic) toxicity studies suggests that F2 obtained from the stem bark of *S. adstringens *is safe at the concentrations tested.

## Figures and Tables

**Table 1:** Acute toxicity of F2 of *Stryphnodendron adstringens* administered orally in mice.

F2 *S. adstringens *	Observed changes									
dose (mg·kg^−1^)	No. of deaths	Diarrhea	Piloerection	Hypoactivity	Hyperventilation	Ptosis	Hypothermia	Motor impairment	Catatonia	Hypnosis/sedation
Control	0	No	No	No	No	No	No	No	No	No
500	0	No	No	No	No	No	No	No	No	No
1,000	0	No	No	No	No	No	No	No	No	No
2,000	2	Reversible after 8 h (2)	Reversible after 72 h (5)	No	No	No	No	No	No	No
3,000	7	Reversible after 8 h (1)	Reversible after 7 d (6)	Reversible after 48 h (4)	Reversible after 48 h (2)	Reversible after 48 h (1)	Reversible after 48 h (1)	Reversible after 48 h (1)	No	No
4,000	9	Reversible after 8 h (6)	Reversible after 7 d (12)	Reversible after 48 h (9)	Reversible after 4 h (2)	No	Reversible after 48 h (4)	No	Reversible after 1 h (1)	No
5,000	11	Reversible after 48 h (6)	Reversible after at 14 d (10)	Reversible after 48 h (5)	Reversible after 12 h (8)	Reversible after 48 h (1)	Reversible after 48 h (4)	Reversible after 1 h (1)	No	Reversible after 12 h (1)

No: no toxic symptoms observed. The numbers in parentheses indicate the number of animals that showed the changes. The mice were observed daily for signs of toxicity (behavioral changes and mortality) for 14 days.

**Table 2:** Effect of F2 of *Stryphnodendron adstringens *on the weights of organs of mice.

Organs	F2 *S. adstringens* (mg·kg^−1^)						
	Control	500	1,000	2,000	3,000	4,000	5,000
Male							
Liver	3.27 ± 0.4	2.57 ± 0.3	2.87 ± 0.2	2.75 ± 0.3	2.58 ± 0.2	2.32 ± 0.3*	2.45 ± 0.6*
Spleen	0.31 ± 0.04	0.24 ± 0.02	0.27 ± 0.06	0.30 ± 0.07	0.20 ± 0.05	0.20 ± 0.03	0.20 ± 0.06
Lungs	0.48 ± 0.01	0.35 ± 0.03	0.40 ± 0.04	0.35 ± 0.04	0.46 ± 0.11	0.39 ± 0.06	0.46 ± 0.16
Heart	0.31 ± 0.03	0.25 ± 0.04	0.27 ± 0.04	0.28 ± 0.04	0.34 ± 0.05	0.29 ± 0.07	0.32 ± 0.04
Kidneys	0.95 ± 0.09	0.67 ± 0.05	0.82 ± 0.05	0.70 ± 0.09	0.81 ± 0.06	0.64 ± 0.10	0.72 ± 0.12
Female							
Liver	2.22 ± 0.3	1.94 ± 0.2	2.04 ± 0.3	1.93 ± 0.3	1.98 ± 0.5	1.92 ± 0.4*	1.68 ± 0.2*
Spleen	0.25 ± 0.04	0.20 ± 0.06	0.28 ± 0.06	0.29 ± 0.09	0.24 ± 0.07	0.26 ± 0.08	0.19 ± 0.03
Lungs	0.38 ± 0.07	0.32 ± 0.06	0.33 ± 0.04	0.36 ± 0.07	0.43 ± 0.15	0.50 ± 0.15	0.47 ± 0.14
Heart	0.20 ± 0.04	0.17 ± 0.04	0.21 ± 0.06	0.24 ± 0.03	0.25 ± 0.07	0.25 ± 0.06	0.19 ± 0.03
Kidneys	0.51 ± 0.06	0.44 ± 0.08	0.49 ± 0.03	0.55 ± 0.07	0.52 ± 0.07	0.51 ± 0.09	0.49 ± 0.03

Values expressed as mean ± DP (standard deviation). ANOVA or Kruskal-Wallis tests. *P < 0.05 compared to control.

**Table 3:** Percentage of weight gain in the animals after 90 days of treatment with the aqueous fraction (F2) of *S. adstringens*.

Groups	Weight gain (%)	
	Male	Female
Control	26.2 ± 3.3	20.8 ± 4.3
10 mg·kg^−1^	22.7 ± 6.2	15.5 ± 4.2
100 mg·kg^−1^	18.4 ± 6.9*	12.8 ± 5.5*
200 mg·kg^−1^	15.2 ± 5.6*	14.8 ± 3.9

*Values are mean ± S.E.M. ANOVA test. *P < 0.05 compared to the control group.

**Table 4:** Biochemical parameters in male and female rats orally treated with F2 of *S. adstringens* for 90 days.

Parameters	Controls	F2 (mg·kg^−1^)		
		10	100	200
Male				
Sodium (mEq·L^−1^)	142.2 ± 5.0	144.5 ± 1.6	142.1 ± 3.3	141.7 ± 2.7
Potassium (mEq·L^−1^)	6.3 ± 0.4	7.3 ± 0.4	10.7 ± 2.4*	7.5 ± 1.2
Glucose (mg·dL^−1^)	122.6 ± 11.6	127.9 ± 12.5	129.4 ± 15.5	123.4 ± 29.0
Triglyceride (mg·dL^−1^)	119.9 ± 23.4	134.5 ± 23.4	95.7 ± 23.5	85.5 ± 28.3*
Cholesterol (mg·dL^−1^)	99.3 ± 13.9	104.5 ± 19.1	97.1 ± 10.2	92.7 ± 21.1
Uric acid (mg·dL^−1^)	1.2 ± 0.2	1.5 ± 0.2	2.7 ± 1.1*	2.0 ± 0.8*
ALP (U·L^−1^)	76.0 ± 18.3	68.4 ± 9.6	84.0 ± 15.8	89.2 ± 13.0
AST (U·L^−1^)	106.7 ± 19.5	148.6 ± 26.3	186.0 ± 47.0*	151.4 ± 67.0
ALT (U·L^−1^)	56.6 ± 7.7	60.9 ± 12.7	90.0 ± 26.1	62.3 ± 13.0
Total protein (g/dL)	6.5 ± 0.6	6.4 ± 0.2	6.8 ± 0.3	6.9 ± 0.5
Total bilirubin (mg·dL^−1^)	0.11 ± 0.02	0.07 ± 0.04	0.11 ± 0.08	0.05 ± 0.04
BUN (mg·dL^−1^)	44.1 ± 3.2	43.9 ± 4.1	50.9 ± 8.9	48.6 ± 8.6
Creatinine (mg·dL^−1^)	0.6 ± 0.2	0.5 ± 0.06	0.4 ± 0.1*	0.5 ± 0.1
*δ*-GT (U·L^−1^)	1.6 ± 0.5	1.7 ± 0.7	2.8 ± 1.6	1.4 ± 0.7

Female				
Sodium (mEq·L^−1^)	132.7 ± 6.0	132.7 ± 5.8	132.4 ± 2.6	136.2 ± 3.2
Potassium (mEq·L^−1^)	6.8 ± 1.1	8.4 ± 1.9	8.6 ± 1.4	6.0 ± 0.3
Glucose (mg·dL^−1^)	116.6 ± 22.9	123.5 ± 14.9	119.5 ± 17.1	131.9 ± 16.4
Triglyceride (mg·dL^−1^)	99.9 ± 20.6	78.5 ± 22.7	58.9 ± 12.5*	72.7 ± 21.3*
Cholesterol (mg·dL^−1^)	98.4 ± 13.3	88.2 ± 11.9	92.0 ± 19.5	96.9 ± 22.2
Uric acid (mg·dL^−1^)	1.4 ± 0.4	1.8 ± 0.8	1.6 ± 0.4	1.1 ± 0.5
ALP (U·L^−1^)	59.5 ± 9.3	57.8 ± 13.6	67.4 ± 16.8	54.5 ± 19.1
AST (U·L^−1^)	173.4 ± 71.9	233.9 ± 90.9	193.5 ± 58.6	136.4 ± 65.9
ALT (U·L^−1^)	59.0 ± 12.9	73.7 ± 20.0	68.5 ± 14.9	51.9 ± 6.1
Total protein (g/dL)	7.4 ± 0.8	7.4 ± 0.2	7.9 ± 0.5	7.3 ± 0.5
Total bilirubin (mg·dL^−1^)	0.13 ± 0.05	0.15 ± 0.08	0.12 ± 0.09	0.13 ± 0.05
(mg·dL^−1^)	49.5 ± 5.9	59.3 ± 12.7	55.2 ± 6.6	45.3 ± 6.8
Creatinine (mg·dL^−1^)	0.6 ± 0.2	0.5 ± 0.1	0.5 ± 0.1	0.6 ± 0.1
*δ*-GT (U·L^−1^)	1.9 ± 0.9	3.3 ± 1.4	3.4 ± 1.4	2.5 ± 0.9

The data are expressed as mean ± SEM. *P < 0.05, compared with control group (ANOVA or Kruskal-Wallis test).

**Table 5:** Hematological results in male and female rats orally treated with F2 of *S. adstringens* for 90 days.

Parameters	Controls	F2 (mg·kg^−1^)		
		10	100	200
Male				
WBC (×10^3^ mm^−3^)	10.6 ± 3.7	7.1 ± 0.6	8.7 ± 2.8	7.2 ± 1.6
Segmented (%)	20.9 ± 8.9	16.0 ± 6.5	15.9 ± 4.9	16.1 ± 4.6
Eosinophil (%)	2.3 ± 0.8	1.9 ± 0.8	1.5 ± 0.5	2.3 ± 0.9
Lymphocyte (%)	66.6 ± 11.8	75.2 ± 9.7	79.3 ± 5.2	78.6 ± 5.6
Monocyte (%)	3.4 ± 0.7	3.1 ± 0.8	3.1 ± 0.5	3.0 ± 0.9
RBC (×10^6^ mm^−3^)	8.16 ± 0.3	8.16 ± 0.4	8.22 ± 0.5	8.50 ± 0.3
Haemoglobin (g/dL)	17.1 ± 0.6	16.0 ± 0.9	16.4 ± 1.1	17.0 ± 0.6
Haematocrit (%)	46.7 ± 1.8	46.3 ± 2.5	46.5 ± 3.2	47.7 ± 2.1
MCV (fL)	57.3 ± 1.2	56.7 ± 1.2	56.5 ± 1.2	55.9 ± 1.4
MCH (pg)	20.9 ± 0.5	20.0 ± 0.4*	19.9 ± 0.5*	20.0 ± 0.4*
MCHC (%)	36.5 ± 0.7	35.6 ± 0.8	35.1 ± 0.3*	35.8 ± 0.8
Platelet (×10^3^ mm^−3^)	679 ± 171	929 ± 108*	961 ± 105*	895 ± 106

Female				
WBC (×10^3^ mm^−3^)	7.2 ± 1.1	10.5 ± 4.3	8.3 ± 2.6	7.9 ± 1.9
Segmented (%)	18.2 ± 8.0	20.2 ± 6.9	14.3 ± 4.3	19.0 ± 9.0
Eosinophil (%)	2.5 ± 0.9	2.1 ± 0.9	2.2 ± 0.9	2.0 ± 1.0
Lymphocyte (%)	76.2 ± 8.3	74.3 ± 8.0	76.5 ± 15.2	75.6 ± 10.3
Monocyte (%)	3.2 ± 0.8	3.1 ± 0.7	2.9 ± 0.8	3.2 ± 1.0
RBC (×10^6^ mm^−3^)	7.35 ± 0.3	7.27 ± 0.6	7.26 ± 0.7	7.5 ± 0.4
Haemoglobin (g/dL)	15.6 ± 0.5	15.3 ± 1.2	15.3 ± 1.6	15.8 ± 0.9
Haematocrit (%)	43.7 ± 1.8	42.9 ± 3.9	45.6 ± 5.3	44.6 ± 2.4
MCV (fL)	59.5 ± 1.1	58.9 ± 1.8	59.9 ± 1.9	59.4 ± 1.1
MCH (pg)	21.2 ± 0.4	21.0 ± 0.5	21.2 ± 1.1	21.1 ± 0.3
MCHC (%)	35.7 ± 0.8	35.6 ± 1.3	35.3 ± 1.9	35.7 ± 0.5
Platelet (×10^3^ mm^−3^)	878 ± 124	884 ± 187	856 ± 168	730 ± 202

The data are expressed as mean ± SEM. *P < 0.05, compared with control group (ANOVA or Kruskal-Wallis test).

## Glossary

F1: Crude extract obtained from the stem bark of *Stryphnodendron adstringens *
F2: Aqueous fraction obtained from the stem bark of *Stryphnodendron adstringens *
LD_50_: Dose that inactivates 50% of individuals.
